# The Long-term Effects of Immersive Virtual Reality Reminiscence in People With Dementia: Longitudinal Observational Study

**DOI:** 10.2196/36720

**Published:** 2022-07-25

**Authors:** Ling-Chun Huang, Yuan-Han Yang

**Affiliations:** 1 Department of Neurology Kaohsiung Municipal Ta-Tung Hospital Kaohsiung Medical University Kaohsiung Taiwan; 2 Department of Neurology Kaohsiung Medical University Hospital Kaohsiung Taiwan; 3 School of Post-Baccalaureate Medicine Kaohsiung Medical University Kaohsiung Taiwan; 4 Neuroscience Research Center Kaohsiung Medical University Kaohsiung Taiwan

**Keywords:** virtual reality, reminiscence, dementia, long-term care

## Abstract

**Background:**

Novel nonpharmacological therapies are being developed to prevent cognitive decline and reduce behavioral and psychological symptoms in patients with dementia. Virtual reality (VR) reminiscence was reported to improve anxiety, apathy, and cognitive function immediately after intervention in individuals at residential aged care facilities. However, its effect on elderly patients with dementia and how long this effect could last remain unknown.

**Objective:**

The aim of this paper is to investigate the effect of immersive VR reminiscence in people with dementia both immediately after and 3-6 months after intervention.

**Methods:**

A pilot study was conducted in 2 dementia care units. VR reminiscence therapy sessions were conducted twice per week for a 3-month period. Cognitive function, global status, depressive symptoms, and caregiver burden were assessed before and immediately after VR intervention in 20 participants. Subsequently, 7 participants were reassessed 3-6 months after the VR intervention. Wilcoxon sign-rank test was used for statistical comparisons of the changes.

**Results:**

There were no significant changes in cognitive function, global status, and caregiver burden immediately after the VR intervention, but there was a significant reduction in depressive symptoms (P=.008). Moreover, compared with the cognitive function immediately after VR, it kept declining 3-6 months after.

**Conclusions:**

Immersive VR reminiscence can improve mood and preserve cognitive function in elderly patients with dementia during the period of the intervention. Studies using a control group and comparing the use of VR with traditional forms of reminiscence should be conducted in the future to confirm and expand on these findings.

## Introduction

Over the past few decades, as the elderly population has rapidly increased, the prevalence of dementia has also continuously increased in high-income countries, and cognitive decline has had a strong impact on society and the economy [1]. Behavioral and psychological symptoms of dementia (BPSD) are frequently noted in patients with dementia, causing distress, reducing the individual’s quality of life, exacerbating cognitive and functional impairment, and increasing caregiver stress [2]. Although pharmacological interventions are recommended for the treatment of cognitive impairment and BPSD in Alzheimer disease (AD) and other types of dementia, polypharmacy may cause side effects in the elderly population. Novel and effective nonpharmacological therapies are therefore required.

Virtual reality (VR) is a computer-generated environment that has been developed in recent years. Using headsets, it can provide a fully immersive, highly engaging, and realistic experience. VR is often used for recreational purposes, but it is increasingly being used in medical training, rehabilitation, and therapy [3-5]. Current research suggests that immersive VR is safe and well tolerated, and it can promote engagement and provide an enjoyable experience for people with dementia [6-8].

Reminiscence therapy, one of the most popular psychosocial interventions, involves the discussion of past activities, events, and experiences, usually with the aid of tangible prompts, such as photographs or music [9]. Reminiscence therapy has been shown to have some positive effects for people with dementia by increasing their quality of life, cognition, communication, and mood, although the noted effects were small [9]. Reminiscence therapy using immersive VR may be more effective as it would be more realistic than traditional reminiscence therapy and could lead to increased engagement. In previous studies, immersive VR reminiscence therapy was shown to reduce anxiety and apathy while improving semantic verbal fluency, immediately after a short intervention program in elderly individuals residing in aged care facilities with different cognitive statuses [10,11]. However, the long-term effects in people with dementia remain unknown.

Therefore, we conducted a study to investigate the immediate and 3-6–month effects of immersive VR reminiscence therapy in people with dementia.

## Methods

### Recruitment

The participants were recruited from 2 dementia care units in Kaohsiung city, Taiwan. Potentially suitable participants were identified by the staff at the dementia care units based on the inclusion and exclusion criteria. The inclusion criteria were (1) those diagnosed with all-cause dementia by experienced physicians, based on the National Institute on Aging and Alzheimer's Association diagnostic criteria [12]; and (2) attended the dementia care units between July 2020 and March 2021. Participants were excluded if (1) they had poor recognition of the VR images even after adjusting the mounting position of the headset; and (2) their cognitive function was too low or their BPSD was too severe, making assessment difficult.

### Ethics Approval

The participants and their relatives were informed of the details of the study prior to their inclusion, and appropriate written informed consent was obtained. The Kaohsiung Medical University Hospital Institutional Review Board (KMUHIRB-SV(II)-20190049) approved the study protocol.

### VR Apparatus

The VIVE Pro VR head-mounted display (HMD) was used to deliver the immersive VR experience to the participants. It is a stand-alone HMD providing stereoscopic vision at a resolution of 2880 x 1600 per eye with a 90 Hz refresh rate. Two controllers were paired with the HMD to enable the participants to interact with the VR environment.

### Preparation of VR Content

**Figure 1:**
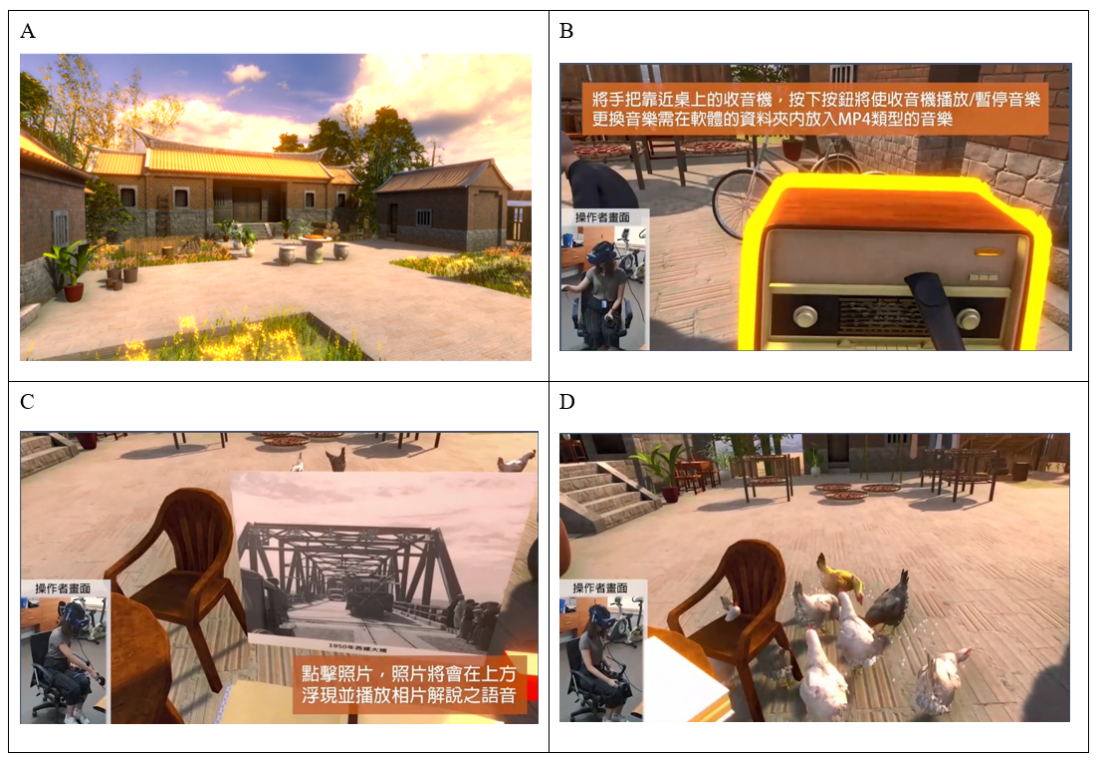
Scenes from the computer generated virtual reality reminiscence. Computer graphics images based on (A) a historical type of residence that was commonly found throughout Taiwan between 1960-1980 AD. (B) The participants could use the controllers to turn on the radio to play music and (C) to open the photo album to browse the photographs with a voice narrating. (D) They could also use the controllers to hold rice to feed chickens, which was traditional in older villages.

Computer graphics VR images were created based on a historical type of residence that was commonly found throughout Taiwan in 1960-1980 AD (Figure 1). Photographs, narration, and music of personal past significance to the participants were provided by the family or caregivers on an individual basis. These materials were transformed into digital forms and integrated into the VR content. The participants could use the controllers to turn on the radio to play the music and to look through the photo album and browse the photographs with a voice narrating the past meaningful situation and events about the photographs. They could also use the controller to hold rice to feed chickens, which was traditional in many old villages.

### Implementation

The VR intervention was administered twice per week over a period of 3 months. The participants viewed and interacted with the VR content for approximately 10 to 12 minutes each time. The HMD images were mirrored to a laptop to enable the researcher to see what the participants were viewing and interacting with. The participants remained seated during the VR intervention to reduce motion sickness when using the HMD and to also minimize the risk of falls in the elderly. During VR intervention, there was a conversation between the researcher and the participant regarding the content being viewed. The participants were provided with the controllers to enable them to interact with the VR space, and the researcher was beside them to assist and guide them when needed.

### Assessment of Questionnaire and Scores

Before and after the VR intervention, the participants were assessed on their cognitive function using the Cognitive Abilities Screening Instrument (CASI), the Mini-Mental State Examination (MMSE), the global status by Clinical Dementia Rating (CDR), and the depressive symptoms by Center for Epidemiological Studies Depression (CESD). Caregiver burden was evaluated using the Zarit Caregiver Burden Interview (ZBI).

### Cognitive Abilities Screening Instrument

The CASI was used to objectively assess overall cognitive function. The CASI has a score range of 0 to 100 and provides a quantitative assessment of attention, concentration, orientation, short-term memory, long-term memory, language abilities, visual construction, list-generating fluency, abstraction, and judgment [13]. Pilot testing has demonstrated its usefulness in monitoring disease progression and in providing profiles of cognitive impairment [13].

### Mini-Mental State Examination

Cognitive impairment was also assessed using the MMSE, which assesses orientation, immediate recall, calculation or attention, delayed recall, naming, repetition, 3-stage command, reading, writing, and constructional praxis. The total score is 30, and a lower score indicates more severe cognitive impairment [14].

### Clinical Dementia Rating

The global status of dementia was defined according to the CDR. The CDR is a widely used clinical staging instrument for characterizing the manifestation and severity of dementia. It is generated from a semistructured interview with the patient and a knowledgeable informant evaluating six cognitive domains (memory, orientation, judgment and problem solving, community affairs, home and hobbies, and personal care). The overall CDR is derived by providing ratings for each of the six domains, where a score of 0 indicates normal; a score of 0.5 signifies uncertain or very mild impairment; and a score of 1, 2, or 3 corresponds to mild, moderate, or severe impairment [15]. The scores for the six domains can then be added together (CDR Sum of Boxes [CDR-SB]).

### Center for Epidemiological Studies Depression

Depressive symptoms were assessed using the CESD, a 20-item self-administered questionnaire. On a 4-point Likert scale ranging from 0 (none) to 3 (5 days or more), the participants were asked to rate the frequency with which each item occurred every week. Higher scores indicate more severe depressive symptoms [16].

### Zarit Caregiver Burden Interview

The ZBI is a well-known self-reporting measure of perceived burden among caregivers. The instrument measures the caregiver’s emotion, psychological health, well-being, social and family life, finances, and degree of control over one’s life. The version used contains 22 items, and each item on the questionnaire is a statement that the caregiver is asked to endorse on a 5-point Likert scale (0: never; 1: rarely; 2: sometimes; 3: quite frequently; and 4: nearly always) [17]. Higher scores indicate higher caregiver burden.

### Statistical Analysis

Data were collected from July 2020 to March 2021. In the results, the data are presented as mean (SD) or as a proportion. Comparisons of scores before and immediately after VR intervention, as well as immediately after and 3-6 months after VR intervention, were performed using the Wilcoxon sign-rank test. Analyses were performed using SPSS 26.0 (IBM Corporation). A 2-tailed *P* value of <.05 was considered to indicate a statistically significant difference.

## Results

### Baseline Demographic Characteristics, Cognitive Function, and Global Status

A total of 25 individuals were enrolled in the study at the beginning; 2 (8%) were excluded because they left the dementia care units, and 3 (12%) were excluded as they were unable to receive a postintervention evaluation due to physical problems. The baseline demographic characteristics, cognitive function, and global status of the 20 participants who completed the study are shown in Table 1. The participants’ mean age was 79.0 (SD 7.8) years; 45% (9/20) were male and 55% (11/20) were female. The mean education level was 10.8 (SD 4.3) years. The mean CASI score was 55.5 (SD 15.6), and the mean MMSE score was 15.4 (SD 5.5). According to CDR, 2/20 (10%) participants had very mild dementia, 15/20 (75%) participants had mild dementia, and 3/20 (15%) participants had moderate dementia.

**Table 1:** Baseline demographic characteristics, cognitive function, and global status of the participants.

Characteristics		Values (N=20)
Age (years), mean (SD)		79.0 (7.8)
**Sex, n (%)**		
	Male	9 (45)
	Female	11 (45)
Education (years), mean (SD)		10.8 (4.3)
CASI^a^, mean (SD)		55.5 (15.6)
MMSE^b^, mean (SD)		15.4 (5.5)
**CDR^c^, n (%)**		
	0.5	2 (10)
	1	15 (75)
	2	3 (15)
CDR sum of boxes, mean (SD)		7.3 (3.0)

^a^CASI: Cognitive Assessment Screening Instrument.

^b^MMSE: Mini-Mental State Examination.

^c^CDR: Clinical Dementia Rating.

### The Immediate Effect of VR Reminiscence

The immediate effects of immersive VR on cognition, global status, depressive symptoms, and caregiver burden are shown in Table 2. There were no significant differences in the MMSE, CASI and its subdomains, CDR-SB, and ZBI scores before and immediately after VR intervention, while the scores for CESD significantly decreased from 6.15 (SD 5.73) to 3.15 (SD 4.26; *P*=.008).

**Table 2:** The immediate effect of virtual reality (VR) reminiscence on cognition, global status, depressive symptoms, and caregiver burden. The comparison of scores before and after VR were evaluated by Wilcoxon sing-rank test.

Characteristics		Before VR, mean (SD)	Immediately after VR, mean (SD)	*P* value
MMSE^a^		15.40 (5.47)	14.95 (5.13)	.48
**CASI^b^**		55.47 (15.64)	54.46 (16.64)	.22
	Remote memory (0-10)	8.75 (1.80)	8.25 (2.51)	.13
	Orientation (0-18)	6.65 (3.86)	6.85 (3.68)	.52
	Attention (0-8)	5.85 (1.43)	5.65 (1.63)	.54
	Concentration (0-10)	5.85 (2.91)	5.95 (3.12)	.77
	Recent memory (0-12)	1.59 (1.55)	1.66 (1.88)	.46
	Fluency (0-10)	3.30 (2.00)	3.40 (2.42)	.95
	Language (0-10)	7.58 (2.39)	7.45 (1.99)	.70
	Abstraction (0-6)	3.00 (1.69)	2.80 (1.32)	.30
	Judgment (0-6)	4.65 (1.27)	4.75 (1.21)	.74
	Visual construction (0-10)	8.25 (2.75)	7.70 (3.33)	.10
CDR^c^ sum of boxes		7.26 (3.00)	7.33 (2.43)	.64
CESD^d^		6.15 (5.73)	3.15 (4.26)	*.008* ^e^
ZBI^f^		34.65 (15.98)	31.20 (14.05)	.14

^a^MMSE: Mini-Mental State Examination.

^b^CASI: Cognitive Assessment Screening Instrument.

^c^CDR: Clinical Dementia Rating.

^d^CESD: Center for Epidemiological Studies Depression.

^e^Significant value is shown in italics.

^f^ZBI: Zarit Caregiver Burden Interview.

### The Long-term Effect of VR Reminiscence

**Figure 2:**
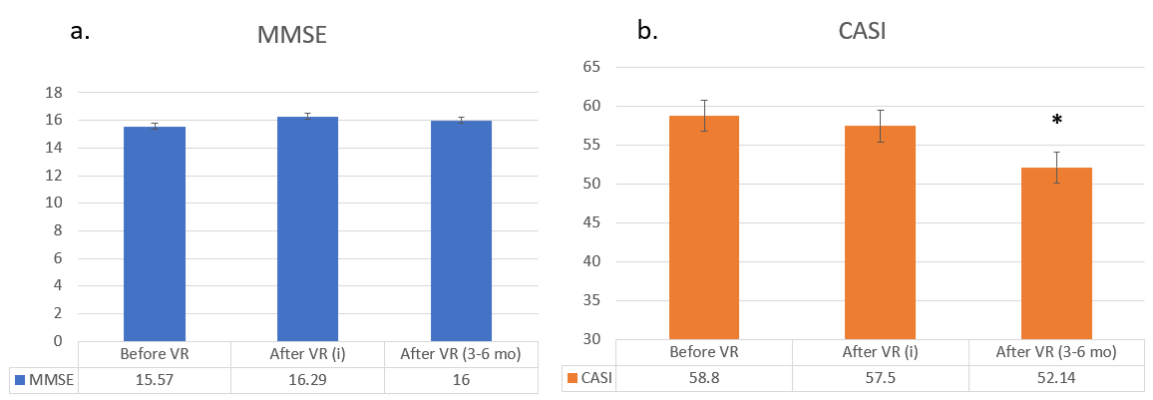
Mini-Mental State Examination (MMSE) and Cognitive Abilities Screening Instrument (CASI) scores before, immediately after, and 3-6 months after virtual reality (VR) reminiscence in 7 participants. Data are shown as mean (SD) for quantitative variables. A comparison of a) MMSE and b) CASI scores before and immediately after VR as well as immediately after VR and 3-6 months after VR were evaluated by Wilcoxon sign-rank test (*P*<.05 is statistically significant); i: immediately.

Among the 20 participants, 7 (35%) were followed up 3-6 months after the VR intervention. No significant changes in MMSE, CASI and CDR-SB were noted before and immediately after VR in these 7 participants; however, the CASI score significantly decreased 3-6 months after VR, compared to immediately after VR (52.14, SD 15.71 vs 57.50, SD 12.40; *P*=.03; Tables 3 and 4; Figure 2).

**Table 3:** Cognition and global status before and immediately after virtual reality (VR) reminiscence in 7 participants. The comparison of scores were evaluated by Wilcoxon sign-rank test.

Characteristics	Before VR, mean (SD)	Immediately after VR, mean (SD)	*P* value
MMSE^a^	15.57 (4.76)	16.29 (4.07)	.67
CASI^b^	58.80 (12.48)	57.50 (12.40)	.50
CDR^c^ sum of boxes	6.50 (1.61)	6.86 (1.28)	.10

^a^MMSE: Mini-Mental State Examination.

^b^CASI: Cognitive Assessment Screening Instrument.

^c^CRD: Clinical Dementia Rating.

**Table 4:** Cognition and global status immediately after and 3-6 months after virtual reality (VR) reminiscence in 7 participants. The comparison of scores were evaluated by Wilcoxon sign-rank test.

Characteristics	Immediately after VR, mean (SD)	3-6 months after VR, mean (SD)	*P* value
MMSE^a^	16.29 (4.07)	16.00 (5.23)	.75
CASI^b^	57.50 (12.40)	52.14 (15.71)	*.03* ^c^
CDR^d^ sum of boxes	6.86 (1.28)	7.93 (2.86)	.31

^a^MMSE: Mini-Mental State Examination.

^b^CASI: Cognitive Assessment Screening Instrument.

^c^Significant value shown in italics.

^d^CDR: Clinical Dementia Rating.

## Discussion

### Principal Results

In this paper, we investigated the potential effects of immersive VR reminiscence therapy in people with dementia, as well as studying how long the effects could last. Although there were no obvious changes in cognition, global status, and caregiver burden after the VR intervention, the depressive symptoms improved significantly after VR therapy. Compared to the CASI scores immediately after VR, the scores after 3-6 months were significantly decreased. In other words, immersive VR reminiscence may improve mood and preserve cognitive function in elderly patients with dementia during the period of intervention. To the best of our knowledge, this is the first study to explore not only the possible effects of immersive VR reminiscence but also the effect period in people with dementia.

### Comparison With Prior Work

Although the use of VR is being developed in many different fields, evidence regarding reminiscence interventions with immersive VR in patients with dementia remains limited. Niki et al [10] found that immersive VR reminiscence could reduce anxiety in the elderly living in a nursing home, without causing serious side effects. However, most of their participants had preserved cognitive function at baseline. Saredakis et al [11] recruited 17 older adults residing in an aged care facility for tailored VR reminiscence and found that it could improve apathy and semantic verbal fluency immediately after the intervention. However, more than half of the participants in that study had no or minimal cognitive impairment. Contrary to these previous studies, we recruited participants who were attending dementia care units to explore the effect of immersive VR reminiscence in individuals with dementia.

People with dementia have a high incidence of depression, which reduces the quality of life for both patients and caregivers and is associated with increased costs and reduced cognition [18]. Although reminiscence therapy may have some benefits in reducing depression in people with dementia, its effectiveness should be tested further [19]. In a multicenter randomized controlled trial, reminiscence therapy failed to improve depressive symptoms in older adults with dementia [20,21], whereas in our study, VR reminiscence significantly reduced depressive symptoms. As an interesting and enjoyable tool, VR intervention may be more effective than traditional reminiscence in improving mood. Further studies comparing VR reminiscence with traditional forms of reminiscence should be conducted in the future.

Based on previous studies, it was not known how long the effects of VR could last. Therefore, we reassessed 7 of the study participants 3-6 months after the VR intervention, and we found that their cognitive function kept declining after discontinuing the intervention. Because more than half of the etiology of dementia is AD, a degenerative disease with an average MMSE decrease of 1.15 points and a CASI decrease of 4.27 points per year [22], the fact that there was no obvious change in cognition during the VR intervention implies that the VR reminiscence therapy may maintain cognition or reduce cognitive decline in people with dementia. Further studies with maintained VR intervention should be conducted to confirm this.

It is reasonable that enough sessions of VR are required to obtain therapeutic effects. In a previous study promoting VR reminiscence in people with dementia, there were no significant changes in the psychological and behavioral symptoms and in the quality of life after a short course of intervention, even though the caregivers assessed the experience as potentially beneficial for most participants [23]. Park et al [24] suggested that reminiscence therapy of more than 8 sessions might be required to obtain any therapeutic effects. Therefore, in our study, the VR intervention was administered twice per week with a period of 3 months.

Our study design was different from that of most previous studies, and our participants could use the controllers to interact with the virtual environment. Interactive VR has been found to increase the sense of presence and to have a positive effect on the immersive experience [25]. Recent research has found that the use of VR in providing interactions may be an alternative way of delivering stimulation to people with dementia who do not participate in other lifestyle activities [26], and interactions can be recorded during the VR experience. In future studies, recording of the participants’ limb movement while they use the controllers could enable analysis of the speed and accuracy of their movements.

### Limitations

There were some limitations to this study. First, there was no control group or a group receiving traditional reminiscence therapy; therefore, the results of this study must be carefully interpreted. Second, the number of subjects was small, especially in the reassessed cases. Studies with larger sample sizes should be conducted in the future to validate our findings. Third, the staff at the dementia care units identified the potentially suitable participants for the study; therefore, the effects may not be applicable to all cases of dementia. Fourth, our VR reminiscence was not totally personalized because computer graphics take a lot of time to create and are expensive. Some VR images were different from those in the subjects’ own memories, and using personalized content has been demonstrated to be more effective than using generic content [27]. To combat this, we collected personal photographs and music from the participants’ past experiences, which could be used to tailor the VR experience. Despite the reported limitations, this study is a pilot study to explore not only the potential effect of immersive VR reminiscence but also how long the effect existed in people with dementia. We also objectively evaluated the possible effects on caregiver burden. During the COVID-19 pandemic, digital therapeutics such as VR are important for facilitating remote health care and for reducing the risk of cluster infections.

### Conclusions

Our study found that immersive VR reminiscence may improve mood and preserve cognitive function in elderly patients with dementia during the period of the intervention. Studies using a control group and comparing the use of VR with traditional forms of reminiscence should be conducted in the future to confirm and develop these findings.

## Abbreviations

AD: Alzheimer disease
BPSD: behavioral and psychological symptoms of dementia
CASI: Cognitive Abilities Screening Instrument
CDR-SB: Clinical Dementia Rating-Sum of Boxes
CDR: Clinical Dementia Rating
CESD: Center for Epidemiological Studies Depression
HMD: head-mounted display
MMSE: Mini-Mental State Examination
VR: virtual reality
ZBI: Zarit Caregiver Burden Interview
